# Genetic Association Analysis of the Functional c.714T>G Polymorphism and Mucosal Expression of Dectin-1 in Inflammatory Bowel Disease

**DOI:** 10.1371/journal.pone.0007818

**Published:** 2009-11-12

**Authors:** Hilbert S. de Vries, Theo S. Plantinga, J. Han van Krieken, Rinke Stienstra, Ad A. van Bodegraven, Eleonora A. M. Festen, Rinse K. Weersma, J. Bart A. Crusius, Ronald K. Linskens, Leo A. B. Joosten, Mihai G. Netea, Dirk J. de Jong

**Affiliations:** 1 Department of Gastroenterology and Hepatology, Radboud University Nijmegen Medical Centre, Nijmegen, The Netherlands; 2 Department of Medicine, Radboud University Nijmegen Medical Centre, Nijmegen, The Netherlands; 3 Nijmegen Institute for Infection, Inflammation and Immunity (N4i), Nijmegen, The Netherlands; 4 Department of Pathology, Radboud University Nijmegen Medical Centre, Nijmegen, The Netherlands; 5 Department of Gastroenterology and Hepatology, VU University Medical Centre, Amsterdam, The Netherlands; 6 Department of Gastroenterology and Hepatology, University Medical Centre Groningen, Groningen, The Netherlands; 7 Department of Pathology, Laboratory of Immunogenetics, VU University Medical Centre, Amsterdam, The Netherlands; 8 Department of Gastroenterology, Saint Anna Hospital, Geldrop, The Netherlands; Charité-Universitätsmedizin Berlin, Germany

## Abstract

**Background:**

Dectin-1 is a pattern recognition receptor (PRR) expressed by myeloid cells that specifically recognizes β-1,3 glucan, a polysaccharide and major component of the fungal cell wall. Upon activation, dectin-1 signaling converges, similar to NOD2, on the adaptor molecule CARD9 which is associated with inflammatory bowel disease (IBD). An early stop codon polymorphism (c.714T>G) in *DECTIN-1* results in a loss-of-function (p.Y238X) and impaired cytokine responses, including TNF-α, interleukin (IL)-1β and IL-17 upon *in vitro* stimulation with *Candida albicans* or β-glucan. The aim of the present study was to test the hypothesis that the *DECTIN-1* c.714T>G (p.Y238X) polymorphism is associated with lower disease susceptibility or severity in IBD and to investigate the level of dectin-1 expression in inflamed and non-inflamed colon tissue of IBD patients.

**Methodology:**

Paraffin embedded tissue samples from non-inflamed and inflamed colon of IBD patients and from diverticulitis patients were immunohistochemically stained for dectin-1 and related to CD68 macrophage staining. Genomic DNA of IBD patients (778 patients with Crohn's disease and 759 patients with ulcerative colitis) and healthy controls (n = 772) was genotyped for the c.714T>G polymorphism and genotype-phenotype interactions were investigated.

**Principal Findings:**

Increased expression of dectin-1 was observed in actively inflamed colon tissue, as compared to non-inflamed tissue of the same patients. Also an increase in dectin-1 expression was apparent in diverticulitis tissue. No statistically significant difference in *DECTIN-1* c.714T>G allele frequencies was observed between IBD patients and healthy controls. Furthermore, no differences in clinical characteristics could be observed related to *DECTIN-1* genotype, neither alone, nor stratified for *NOD2* genotype.

**Conclusions:**

Our data demonstrate that dectin-1 expression is elevated on macrophages, neutrophils, and other immune cells involved in the inflammatory reaction in IBD. The *DECTIN-1* c.714T>G polymorphism however, is not a major susceptibility factor for developing IBD.

## Introduction

Inflammatory bowel disease (IBD), is an idiopathic, chronic, relapsing inflammatory disorder of the gastrointestinal tract. It is commonly accepted that IBD is caused by an exaggerated cell mediated immune response to intestinal microbiota in genetically susceptible individuals[1], [2]. IBD mainly involves two distinct diseases, which show some overlap: Crohn's disease (CD) and ulcerative colitis (UC). Genetic susceptibility is more pronounced in CD compared to UC[3]. Several susceptibility loci for developing CD have been identified in the past decades including the *NOD2* gene within the *IBD1* locus[4]. The established association of *NOD2 (CARD15)* with CD emphasizes the important role of the intestinal microbiota in the pathogenesis of CD, since NOD2 acts as an intracellular pattern recognition receptor (PRR) recognizing bacterial peptidoglycans[5], [6].

Dectin-1 (*CLEC7A*) is a pattern-recognition receptor expressed by myeloid cells which specifically recognizes β-(1,3)-glucan, a polysaccharide and component of the fungal cell wall. As a result, dectin-1 is involved in recognition of fungi such as *Candida albicans* and *Aspergillus fumigatus*. Upon activation, dectin-1 recruits spleen tyrosine kinase (Syk) which in turn activates NF-κB, requiring the adaptor molecule Caspase Activating Recruitment Domain 9 (CARD9), a key adaptor for non-Toll Like Receptor (TLR) signal transduction[7]. Although not the exclusive pathway, CARD9 also has a critical function in NOD2-mediated activation of the kinases p38 and Jnk, required for the production of pro-inflammatory cytokines in innate immune responses to intracellular pathogens[8]. LeibundGut-Landmann et al. showed that dectin-1-Syk-CARD9 signaling induces dendritic cell (DC) maturation and secretion of pro-inflammatory cytokines like interleukin (IL)-6, TNF-α, IL-17 and IL-23[9]. Furthermore, Zhernakova et al. identified *CARD9* as a susceptibility locus for IBD[10]. Recently, the *DECTIN-1* polymorphism c.714T>G on chromosome 12p13 has been described, with a transition from a tyrosine to an early stop codon on amino acid position 238 (p.Y238X)[11]. The functional consequence of this polymorphism is a complete loss-of-function, and immune cells expressing this truncated protein produce significantly less cytokines, including TNF-α, IL-1β and IL-17, upon *in vitro* stimulation with β-glucan or *Candida albicans* [12].

Th17 responses are considered to be involved in the pathogenesis of auto-immune diseases. This T cell subset appears to play a role in the etiology of CD since IL-17 is up-regulated in the intestine of IBD patients[13]. Interestingly, both NOD2 and dectin-1 are shown to be capable of inducing Th17 responses after activation[9], [14]. In this respect, the *DECTIN-1* c.714T>G polymorphism could influence the Th17 response towards fungi such as *Candida albicans*, a commensal microorganism of the gastrointestinal tract. *Candida albicans is* also one of the immunogens for developing antibodies against *Saccharomyces cerevisiae* (ASCA), which are regularly observed in patients with CD[15], [16].

Taking together the data from various studies, a dysregulation of the immune response to the commensal *Candida albicans* through dectin-1 and IL-17 release might play a role in the pathogenesis of CD.

As stated above, activation of NOD2 and dectin-1 leads to signaling through a shared pathway (CARD9). The importance of this pathway in CD is demonstrated by the fact that mutations in *NOD2* and *CARD9* and the presence of circulating ASCA, are associated with CD. Since the c.714T>G polymorphism within *DECTIN-1* results in a loss of function, we hypothesized that this polymorphism could be potentially protective against developing IBD. Therefore, we aimed to elucidate the role of the *DECTIN-1* c.714T>G (p.Tyr238X) polymorphism in patients with IBD, focusing on the occurrence and the clinical severity of IBD.

## Methods

### Patients

Patients with a diagnosis of IBD were recruited from the outpatient clinics of three university hospitals in the Netherlands: Radboud University Nijmegen Medical Centre (CD: n = 161, UC: n = 212), VU University Medical Centre Amsterdam (CD: n = 177, UC: n = 148) and the University Medical Centre Groningen (CD: n = 308, UC: n = 214), and one regional hospital: St. Anna Hospital, Geldrop (CD: n = 132, UC: n = 185). Healthy controls were recruited at the Radboud University Nijmegen Medical Centre and at the University Medical Centre Groningen (n = 772).

Diagnosis of IBD was based on accepted clinical, endoscopic, radiological and histological findings[17]. Clinical data on patients were retrieved by retrospective collection from patients' clinical charts. Clinical data on patients from the VU University Medical Centre were collected prospectively. The following data were obtained from patients with CD: age, age at diagnosis, gender, familial or sporadic IBD, disease localization and behavior of disease (according to the Vienna classification[18]), extra-intestinal manifestations, peri-anal disease, and surgery for CD. For patients with UC, the following data were obtained: age, age at diagnosis, disease location (according to the Montreal classification[19]), familial or sporadic IBD, extra intestinal manifestations, surgery for UC, and occurrence of colorectal cancer.

The ethical committee of region Nijmegen and Arnhem reviewed and approved the protocol under number CWOM-nr 9804-0100. Verbal informed consent was obtained from each patient before study participation in agreement with the approval and all samples were anonymized. Given the fact that all research data were anonymously collected, at least verbal informed consent was needed according to national regulations. Therefore, since written informed consent was not required, no written informed consent procedure was introduced at time of data collection.

### Genotyping of c.714T>G polymorphism in DECTIN-1

Genomic DNA was isolated from peripheral venous blood using standard techniques and stored at 4°C. Genotyping of the c.714T>G (p.Y238X) polymorphism in exon 6 of the *DECTIN-1* gene in the patient and healthy control groups from Nijmegen, Amsterdam and Geldrop was performed by applying the predesigned TaqMan SNP assay C_33748481_10 (rs 16910526) on the 7300 ABI Real-Time PCR system (both from Applied Biosystems, Foster City, CA, USA) using 96-well plates. Genotyping of the IBD cohort and healthy controls from Groningen, was performed at the Department of Genetics, UMC Groningen, the Netherlands, applying the same predesigned TaqMan SNP assay, using the 7900 ABI Real-Time PCR system (Applied Biosystems, Foster City, CA, USA). The patient and control DNA samples from Groningen were processed in 384-well plates and each plate also contained 16 genotyping controls (4 duplicates of the Centre d'Etudes du Polymorphisme Humain (CEPH) DNA samples 123002,102405,090203 and 081505). For all polymorphisms we obtained >99.8% concordance between our CEPH genotype data and the CEU (European ancestry) data available from HapMap.

### Genotyping of NOD2 variants

Data on the three common *NOD2* variants were available for CD patients from Groningen and Nijmegen (p.Arg702Trp; n = 437, p.Gly908Arg; n = 446, p.Leu1007ProfsX2; n = 436). Genotyping of these three *NOD2* variants (c.2104C>T (p.Arg702Trp), c.2722G>C (p.Gly908Arg), c.3019_3020insC (p.Leu1007ProfsX2)) has been described before[20].

### Immunohistochemical staining

Dectin-1 and CD68 protein expression were evaluated by immunohistochemical staining in paraffin embedded normal and inflamed colon tissue of five IBD patients and 4 patients with diverticulitis, all homozygous for the *DECTIN-1* wild-type allele (T/T). The applied primary antibodies were a monoclonal mouse-anti-human dectin-1 antibody (MAB 1859, purchased from R&D Biosystems, Minneapolis, MN, USA), used in a concentration of 5 µg/ml and a monoclonal mouse-anti-human antibody against CD68 (MCA1815T, purchased from AbD Serotec, Oxford, UK) which was diluted 100 times before use. After overnight incubation with the primary antibody, the tissue sections were incubated for 1 hour with a secondary antibody after washing with PBS. Subsequently the staining was visualized by applying ABC complex and DAB solution. Sections were counterstained with haematoxylin.

### Statistics

Statistical analysis was performed by using SPSS statistical software, version 16.0 (SPSS Inc., Chicago, IL). Controls and IBD patients were tested for Hardy Weinberg equilibrium. Allele frequencies were compared between patients and controls using the χ^2^ test. *P-*values were obtained by comparing individuals carrying at least one *DECTIN-1* G allele (G/G genotype and T/G genotype) with wild-type individuals (T/T genotype). Continuous variables were compared using Student t-tests. Strength of association between genotype and phenotype is given as odds ratio with 95% confidence interval (CI). Statistical interaction between *NOD2* variants and the c.714T>G polymorphism regarding clinical characteristics, was investigated by comparing patients carrying a *NOD2* susceptibility allele in combination with carrying one or two copies of the *DECTIN-1* G allele, to patients not bearing any of these *NOD2* or *DECTIN-1* minor alleles. This combined analysis for c.714T>G and *NOD2* was performed for each of the three *NOD2* susceptibility alleles. A *P*-value <0.05 was considered significant.

## Results

### Protein expression of dectin-1 in intestinal tissue

**Figure 1:**
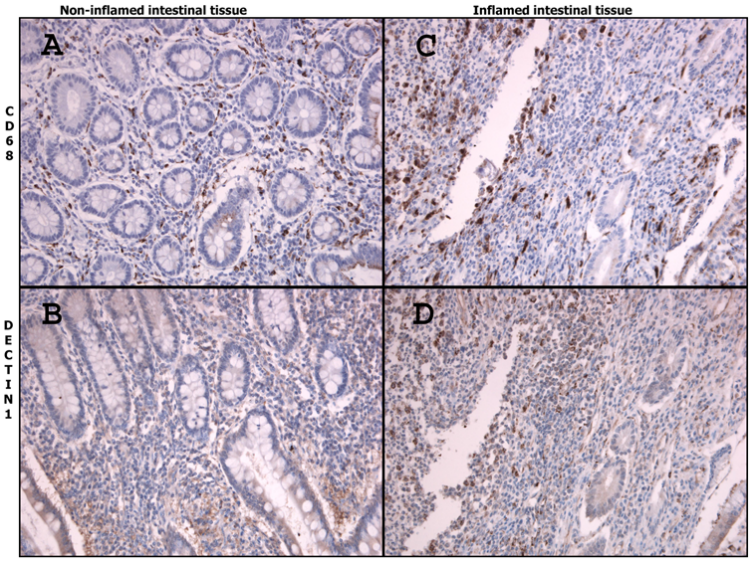
Representative immunohistochemical staining of DECTIN-1 and CD-68 in inflamed and non-inflamed intestine of the same specimen in Crohn's disease (250× magnified). Macrophages are present in non-inflamed intestinal tissue but are present in increased numbers in inflamed tissue (pictures A and C). The expression of DECTIN-1 is increased in inflamed intestinal tissue compared to non-inflamed intestinal tissue (pictures B and D).

**Figure 2:**
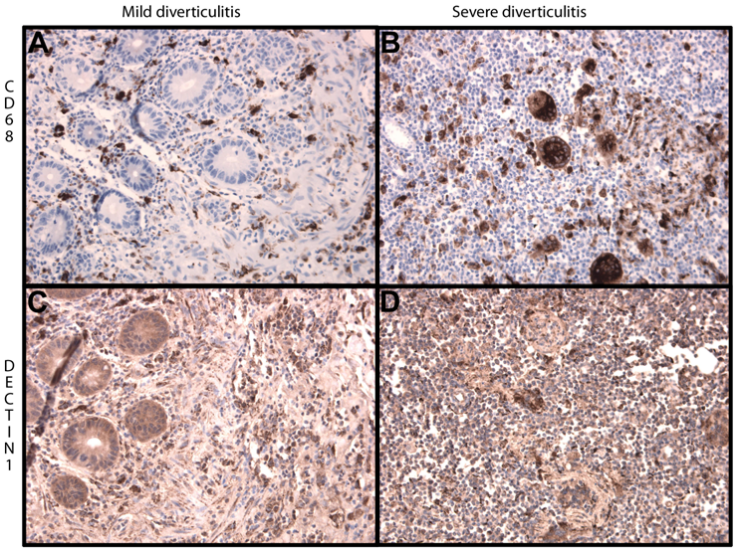
Representative immunohistochemical staining of dectin-1 and CD68 in mild and severe diverticulitis (250× magnified). Macrophages are present in intestinal tissue of mild diverticulitis but are present in increased numbers in severe diverticulitis (pictures A and B). Also, the expression of dectin-1 is increased in severe diverticulitis compared to mild diverticulitis (pictures C and D).

Dectin-1 and CD68 staining was performed on matched intestinal tissue samples from five IBD patients, either inflamed or non-inflamed, as depicted in Figure 1 and 2. Dectin-1 expression is mainly present on macrophages as showed by staining for CD68 (Figure 1). Furthermore, dectin-1 also appears to be weakly expressed on neutrophils, the membrane of endothelial and epithelial cells and in the submucosal neuronal plexus of Meissner (not shown). Dectin-1 appeared to be up-regulated within inflamed colon tissue due to increased expression of dectin-1 on inflammatory cells and increased influx of inflammatory cells (Figure 1). In order to test whether this increased expression of dectin-1 is IBD specific, additional staining was performed on matched intestinal tissue samples from 4 patients with diverticulitis. As shown in Figure 2, increased expression of dectin-1 was also observed in patients with severe diverticulitis compared to mild diverticulitis. As is true for patients with IBD, expression of dectin-1 is mainly present on macrophages as shown by CD68 staining.

### Distribution of the DECTIN-1 c.714T>G polymorphism in IBD patients and healthy controls

Characteristics of the study population and healthy controls are depicted in Table 1. Genotype frequencies of healthy controls were in Hardy Weinberg equilibrium. Carriage of at least one copy of the G allele of the *DECTIN-1* polymorphism was 14% in the CD, 14% in the UC and 17% in the healthy control group. The frequency of the G allele was 9.8% in the healthy controls, 8.1% in CD patients and 7.7% in UC patients. Overall, no significant differences were observed between patients with IBD and healthy controls regarding allele frequencies of the *DECTIN-1* polymorphism. However, a slight trend towards association of homozygosity for the *DECTIN-1* G allele with IBD was observed (G allele frequency of 1.0% in healthy controls vs. 0.5–0.6% in CD and UC, Table 1).

**Table 1:** Distribution of genotypes of wild-type, heterozygous and homozygous individuals for the c.714T>G polymorphism.

*DECTIN-1* status	Controls		Crohn's disease		*P*-value*	Ulcerative colitis		*P*-value*
Total cohort, number	772	(100%)	778	(100%)		759	(100%)	
T/T	642	(83.2%)	667	(85.7%)	0.16	655	(86.3%)	0.09
T/G	122	(15.8%)	106	(13.6%)		100	(13.2%)	
G/G	8	(1.0%)	5	(0.6%)		4	(0.5%)	

Values are presented as absolute numbers (percentages).

*Healthy controls versus patients with IBD; carriers of the mutant allele (T/G and G/G) were compared to wild-types (T/T).

### Correlation of the DECTIN-1 c.714T>G polymorphism with clinical characteristics of IBD patients

Patients with CD carrying one or two copies of the *DECTIN-1* G allele were compared to patients with the wild-type genotype T/T with regard to age at diagnosis, gender, family history of IBD, localization of disease and disease behavior, extra intestinal and peri-anal disease and surgery related to CD (Table 2). Patients with UC were likewise compared according to the *DECTIN-1* genotypes regarding age at diagnosis, gender, localization of disease, extra-intestinal disease, development of malignancies, surgery related to UC and a positive family history for IBD (Table 3). No statistical significant associations were observed between the c.714T>G polymorphism and specific phenotypes.

**Table 2:** Association between *DECTIN-1* genotypes and clinical characteristics in a subset of Crohn's disease patients from whom detailed phenotypic data were available (N = 778).

Characteristic	Total cohort CD	(%)	T/T	(%)	T/G	(%)	G/G	(%)	Odds ratio*	95% CI*	
Mean age at diagnosis, yr (SD)			29.58 (±12.29)		29.37 (±12.64)		30.76 (±17.32)				
Male gender (%)	261	(33.5)	222/667	(33.3)	37/106	(34.9)	2/5	(40.0)			
Familial IBD (N = 631)	131	(20.8)	107/588	(20.3)	24/101	(24.0)	0/5	(0)	1.32	0.78	2.17
Localization (Vienna Classification) (%) (n = 778)											
L1: ileum	196		164/667	(24.6)	32/106	(30.2)	0/0	(0)	1.24	0.80	1.94
L2: colon	194		163	(24.2)	28	(26.4)	3	(60.0)	1.20	0.76	1.88
L3: ileocolonic	388		340	(51.0)	46	(43.4)	2	(40.0)	0.73	0.49	1.10
L4: upper disease	43		36	(5.4)	7	(6.6)	0	(0)	1.18	0.51	2.72
Disease behavior (Montreal classification) (%) (n = 776)											
B1: non structuring, non penetrating (%)	291	(37.5)	250/665	(37.6)	39/106	(36.8)	2/5	(40.0)	0.97	0.64	1.47
B2: structuring	215	(27.7)	187	(28.1)	27	(25.5)	1	(20.0)	0.86	0.54	1.37
B3: penetrating	270	(34.8)	228	(34.3)	40	(37.7)	2	(40.0)	1.16	0.77	1.77
Extraintestinal disease (%) (n = 750)	151	(20.1)	125/642	(19.5)	26/103	(25.2)	0/5	(0)	1.31	0.81	2.13
Perianal disease (%) (n = 643)	177	(27.5)	149/548	(27.2)	26/90	(28.9)	2/5	(40.0)	1.12	0.69	1.81
Surgery (n = 774)	411	(53.1)	355/664	(53.5)	54/105	(51.4)	2/5	(40.0)	0.90	0.60	1.35

Values are presented as absolute numbers (percentages).

*Carriers of the mutant allele (T/G and G/G) were compared to wild-types (T/T).

**Table 3:** Association between *DECTIN-1* genotypes and clinical characteristics in a subset of ulcerative colitis patients from whom detailed phenotypic data were available (N = 759).

Characteristic	Total cohort UC	(%)	T/T	(%)	T/G	(%)	G/G	(%)	Odds ratio*	95% CI*	
Age at diagnosis (SD)			36.3 (±14.4)		33.9 (±12.9)		35.5 (±10.4)				
Male gender	401	(52.8)	346/655	(52.8)	52/100	(52.0)	3/4	(75.0)			
Localization (Montreal) (n = 721)											
E1 (Proctitis)	124	(17.2)	110/623	(17.7)	14/95	(14.7)	0/3	(0)	0.78	0.43	1.42
E2 (Left sided)	245	(44.0)	212	(34.0)	33	(34.7)	0	(0)	0.98	0.63	1.54
E3 (Extended/pancolitis)	352	(48.8)	301	(48.3)	48	(50.5)	3	(100)	1.16	0.76	1.79
Extraintestinal disease (n = 228)	42	(18.4)	33/183	(18.0)	8/43	(18.6)	1/2	(50)	1.14	0.50	2.59
Surgery (n = 759)	145	(19.1)	120/655	(18.3)	24/100	(24.0)	1/4	(25.0)	1.41	0.86	2.31
Malignancy (n = 384)	2	(0.5)	1/329	(0.3)	1/53	(1.9)	0/2	(0)	6.07	0.37	98.57
Family diagnosis of IBD (n = 547)	81	(14.8)	72/472	(15.3)	9/72	(12.5)	0/3	(0)	0.76	0.36	1.59

Values are presented as absolute numbers (percentages).

*Carriers of the mutant allele (T/G and G/G) were compared to wild-types (T/T).

### The DECTIN-1 c.714T>G polymorphism stratified by NOD2 status and clinical characteristics of IBD patients

CD patients carrying one or two copies of the G allele of the *DECTIN-1* gene were stratified by *NOD2* status. A *NOD2* risk genotype was defined as carrying at least one of the three common *NOD2* disease susceptibility alleles (c.2104C>T (p.Arg702Trp), c.2722G>C (p.Gly908Arg), c.3019_3020insC (p.Leu1007ProfsX2)). Combinations of *NOD2* risk carriers and *DECTIN-1* c.714T>G carriers were compared to patients not bearing any of these *NOD2* or *DECTIN-1* minor alleles, regarding clinical characteristics. No statistical significant interaction between *DECTIN-1* c.714T>G and one of the *NOD2* variants was observed (data not shown).

## Discussion

Signaling through dectin-1, known for its recognition of the fungal component β-glucan, has been described to be involved in several immunological pathways. Dectin-1 amplifies pro-inflammatory cytokine production induced by TLR2 and TLR4, and primes Th1, Th17 and cytotoxic T cell responses induced by dendritic cells[9], [21]. The *DECTIN-1* c.714T>G polymorphism results in a loss-of-function of dectin-1, and we hypothesized that this polymorphism could be potentially protective in either the susceptibility to or the disease severity of IBD.

As shown by immunohistochemical staining of intestinal tissue, dectin-1 is mainly present on macrophages, but also weakly on epithelial and endothelial layers of the intestine. Similar findings in mice have been demonstrated by Wong and co-workers, who demonstrated that dectin-1 is mainly expressed on populations of myeloid cells (monocyte/macrophage and neutrophil lineages)[22]. In addition, they demonstrated that dectin-1 is also expressed in the Peyer's patches and along the lamina propria of the mouse intestine[23], [24]. Interestingly, dectin-1 expression appeared to be elevated in inflamed intestinal tissue compared to normal tissue, due to the increased infiltration of immune cells and increased dectin-1 expression on the cell membrane of immune cells.

However, intestinal expression of dectin-1 did not appear to be disease specific but rather dependent on influx of macrophages. In fact, expression of dectin-1 was also present in intestinal samples from patients with diverticulitis. Increased infiltration of macrophages in severe diverticulitis showed an increased expression of dectin-1, compared to mild diverticulitis which is accompanied by less infiltration of macrophages.

Cohorts of CD (n = 778) and UC (n = 759) patients were screened for the *DECTIN-1* c.714T>G polymorphism and compared to a group of healthy subjects (n = 772). Subsequently, these genetic data were correlated with clinical parameters reflecting disease severity. This analysis revealed no statistical significant association between the prevalence of the *DECTIN-1* c.714G allele and IBD, neither in disease occurrence nor in disease severity. However, one can observe that homozygous individuals bearing the *DECTIN-1* polymorphism were twice as frequent in healthy controls compared to IBD patients. This may suggest that complete absence of dectin-1 function could protect against IBD. It is important to realize that the only two dectin-1 isoforms capable of binding β-glucans (isoforms A and B) are structurally equally affected by the *DECTIN-1* c.714T>G polymorphism[25]. The occurrence of splicing isoforms with residual function could therefore be excluded. The potential mechanism of protection are likely to include the lower production of pro-inflammatory cytokines, including IL-17, in the individuals with defective dectin-1. In these series, statistical power to preclude any functional difference is insufficient due to the low prevalence of homozygous individuals. Additional studies in homozygous and heterozygous subpopulations are needed to confirm the reported observations.

All together, the reports of the association of mutations within *NOD2* and *CARD9* in patients with CD, the presence of ASCA, and the shared signaling pathway of dectin-1 and NOD2, points toward a possible link between NOD2 and dectin-1[26]. Since mutations in *NOD2* in CD patients are associated with ileal involvement and increased need for surgery and stricturing disease[27], a potential interaction between *NOD2* mutations and the *DECTIN-1* c.714T>G polymorphism with regard to phenotypical characteristics was investigated. However, no statistical interaction could be demonstrated (data not shown).

Our data demonstrate that dectin-1 expression is elevated on macrophages, neutrophils, and other immune cells involved in the inflammatory reaction in IBD. The *DECTIN-1* c.714T>G polymorphism is not a major susceptibility factor for protection against IBD, although a trend towards a lower frequency of the polymorphism in CD and UC cohorts was observed, in particular in the number of individuals homozygous for the *DECTIN-1* polymorphism. These genetic findings warrant further investigation of this pathogenetic pathway.
